# Artificial Respiration

**Published:** 1908-07-11

**Authors:** 


### The Hospital

A Journal of

The Medical Sciences and Hospital Administration.

New Series. No. 70, Vol. III. [No. 1142, Vol. XLIV.] SATURDAY, JULY 11, 1908.

### ARTIFICIAL RESPIRATION.

For many years the accepted method of performing artificial respiration on those in whom that function is suspended has been that associated with the name of the late Dr. Silvester, of Clapham. This method is so well known to all members of the medical profession that it is not necessary to describe its details here. Suffice it to say that a committee of the Medico-Chirurgical Society reported in its favour as long ago as 1862, and that it was officially adopted by the Royal Humane Society a few years later. Some five years ago the principles upon which this method is founded were assailed by Professor Schäfer, who proposed an alternative scheme of his own. This scheme was adopted by another Committee of the Medico-Chirurgical Society, and last year by the Royal Life Saving Society also; but it has been a good deal criticised by those who adhere to the Silvester system. Dr. Arthur Keith does valuable service, therefore, by a dispassionate survey of the whole matter * based upon investigations both in the dissecting-room and on living subjects.

The essentials of the Schäfer method are the adoption of the prone as opposed to the supine position advocated by Silvester, and the production of the expiratory movement by pressure applied over each side of the lower part of the thorax; inspiration is induced, when this pressure is relaxed, by the natural elastic recoil of the ribs. The usual precautions as to the patency of the air-way, loosening of clothing, and so forth, one may take for granted, as common to all methods. The advantages claimed for the prone position and the Schäfer system of compression are very briefly these:—The tongue falls forward, and is less likely to obstruct the airway; water (in the case of the apparently drowned) is at once squeezed out; a single operator can conduct the process unaided for an indefinite time; the plan is highly efficient in practice; and, lastly, compression of the lungs is a better stimulant to the respiratory centre than is expansion. In examining these claims by experiment, the first point which struck Dr. Keith was that it is impossible to be both conscious and passive when someone suddenly thrusts a weight of about 60 lbs. over the loin; and therefore all deductions from experiments on conscious subjects contain an element of fallacy. He had previously tested the air exchange obtained by the two conflicting methods on a healthy conscious student, and found a slight advantage in the Schäfer method; but on repeating the experiment on the same subject when unconscious, presumably during anæsthesia, though we are not directly told so, he found the Silvester method more efficient in this respect. Subsequently it occurred to him to measure the difference in the elasticity of the thorax in the prone and supine positions, and this was done on five dead and ten living subjects. In the prone position there is already superincumbent on the sternum and ribs a considerable weight due to the thoracic contents, vertebral column, and so forth; and it was, in fact, determined that in this attitude there is a very considerable loss of elasticity, which is greater in the dead than in the conscious living subject. So far, then, Dr. Keith's results would apparently favour the old plan; but he points out that there are other factors to be considered besides the mere recoil of the thorax. As a result of practical experience on his own body, he concludes that there is a bend forwards of the lumbar spine during compression in the Schäfer system which is accompanied by a compensatory bend back of the thoracic spine, and also that the forcible compression of the abdominal viscera drives up the diaphragm; now both these effects must aid expiration, and their reversal must follow the removal of compression. He thinks, too, that in the hands of untrained operators the Schäfer method is less likely to be ineffectively applied than is the other. Still he sums up by saying that "There is much to be done to put the matter on a sound scientific foundation."

From the point of view of the instruction of the public, artificial respiration is chiefly of use in restoring the apparently drowned; whereas by members of the medical profession it is probably more frequently made use of in such emergencies as poisoning by chloroform, morphia, and other drugs, and in various asphyxial conditions, of which immersion is only one. It must not be forgotten, therefore, that a method which by reason of its simplicity or its special adaptation to the needs of the immersed is especially useful to the lay public is not necessarily the best in all circumstances for the medical man. Only a few days ago an actual

* "London Hospital Gazette," June 1908.

happening has proved very forcibly the truth of this: An operating surgeon was engaged in gingerly removing a pus-distended but unruptured appendix, when without warning the anæsthetist demanded artificial respiration for the patient, and hastily reversed the latter's position; the result of this was the rupture of the appendix and the distribution of its purulent contents about the field of operation, a *contretemps* which might have been, but fortunately was not, followed by the most serious results. Whether or not the Schäfer method ultimately supplants the older procedure of Silvester—and this the practical experience of the next few years will decide—as the routine to be taught to members of Ambulance Classes and First-Aid Societies, the medical practitioner must always consider the circumstances of the individual case, and apply to them his trained intelligence in deciding what to do. One more point: when a layman of very slight training is confronted by circumstances in which artificial respiration is called for, he always applies his knowledge with great energy. There seem distinct possibilities of serious damage to the abdominal and thoracic viscera if forcible compression be applied to a woman or child in the prone position by a man of powerful physique, especially as such men are often totally ignorant of their own strength. Dr. Keith comments on the sensations he experienced in the region of his liver when being treated by the Schäfer method, and the warning note which he sounds in this connection is certainly not by any means out of place. The difficulty is always to persuade the public to refrain from unnecessary intervention, and every police surgeon could relate instances of prolonged artificial respiration applied to patients who were, but for that, making perfectly successful efforts to breathe for themselves. Therefore any method put into the hands of the laity must, if possible, be free from ill effects when applied with more vigour than discretion.

